# Photoselective sharp enucleation of the prostate with a front-firing 532-nm laser versus photoselective vaporization of the prostate in the treatment of benign prostatic hyperplasia: a randomised controlled trial with 1-year followup results

**DOI:** 10.1186/s12894-022-01129-x

**Published:** 2022-11-07

**Authors:** Zhengchao Liu, Zhipeng Chen, Dishi Yan, Tao Jiang, Jian Fu, Jun Zheng, Yuanxiu Zhou, Zhansong Zhou, Wenhao Shen

**Affiliations:** 1 Urological institution of the People’s Liberation Army, First Affiliated Hospital to Army Medical University, Third Military Medical University), 400037 Chongqing, China; 2 Department of Anesthesiology, Daping Hospital, Third Military Medical University, Chongqing, China

**Keywords:** Benign prostatic hyperplasia, Laser, Enucleation, Vaporization

## Abstract

**Background:**

We designed a new surgical procedure to treat benign prostatic hyperplasia(BPH). In order to verify its effectiveness and safety, we constructed this randomized controlled trial to compare the efficacy of our innovative enucleation technique- photoselective sharp enucleation of the prostate (PSEP), with a front-firing 532-nm laser and the traditional technique-photoselective vaporization of the prostate (PVP) in the treatment of BPH.

**Methods:**

A total of 154 consecutive patients diagnosed with bladder outlet obstruction secondary to BPH in our center from June 2018 to April 2019 were randomly divided into the PSEP group (n = 77) and the PVP group (n = 77) and were treated surgically with either PSEP or PVP. All patients were assessed preoperatively and followed up at 1, 6, and 12 months postoperatively. The international prostate symptom score,quality-of-life score, postvoid residual urine volume, maximum urine flow rate, prostate volume, prostate-specific antigen, and adverse events were compared.

**Results:**

The lower urinary tract symptoms in both groups were significantly improved compared with the baseline at 1, 6, and 12 months postoperatively. The PSEP and PVP groups had an equivalent International Prostate Symptom Score, quality-of-life score, postvoid residual urine volume, maximum urine flow rate, prostate-specific antigen at each follow-up (P > 0.05). The median operative time in the PSEP group was significantly shorter than that in the PVP group (35 min vs. 47 min, P < 0.001). At 6 and 12 months after surgery, the median PV in the PSEP group was smaller than that in the PVP group (P < 0.05). Complication rates were comparable between the groups.

**Conclusion:**

Both PSEP and PVP can achieve good efficacy and safety in the treatment of BPH. PSEP can remove more tissue than PVP and is associated with higher efficiency. In addition, PSEP eliminates the problem of lack of tissue samples associated with PVP.

**Trial registration:**

Chinese Clinical Trial Registry, identifie:ChiCTR1800015867, date:25/04/2018.

## Background

Photoselective vaporization of the prostate (PVP) has been used in the treatment of benign prostatic hyperplasia (BPH) for more than 20 years, and its safety and effectiveness have been well demonstrated [1]. It is recommended as the first-choice treatment for patients taking oral antiplatelet/anticoagulant drugs because of the selective absorbing of the laser energy by hemoglobin [2]. However, the limitations of PVP have attracted attention in recent years. One limitation is the challenge of completely vaporizing the transition zone in large-volume prostates. Although prostates > 80 ml can be treated with PVP, these cases are associated with a long operative time, extra fibers, and a high reoperation rate due to incomplete vaporization [3–5]. Moreover, there are no pathological specimens after PVP, which may lead to missed diagnosis of prostate cancer.

These limitations were partially overcome by a procedure called GreenLight laser enucleation of the prostate (GLEP), which used a side-firing fiber for enucleation. GLEP mainly relies on blunt mechanical separation along the capsular plane using the endoscopic sheath, and the laser is mainly used for hemostasis and vaporization of adherences between the capsule and the hyperplastic adenoma. Compared with PVP, GLEP retains tissue specimens, removes hyperplastic glands more thoroughly, and improves surgical efficiency [6]. However, extra fibers are still needed for patients with large prostates [7]. Notably, GLEP is associated with a higher incidence of postoperative stress urinary incontinence (3.4–25%) [6, 8], rate of capsule perforation (11.6–28%)[6, 9], and rate of auxiliary transurethral resection of the prostate (TURP) for hemostasis (10–46%) [6, 9, 10]. The study of Elshal et al. showed that GLEP had a lower prostate size reduction (43.1% vs. 74.3%) and a higher rate of auxiliary TURP for hemostasis (24.5% vs. 4%) than holmium laser enucleation of the prostate (HoLEP) [10], which is usually considered as an energy-based (sharp) enucleating transurethral procedure.

Energy-free (blunt) enucleation may proceed smoothly when a clear edge is present between the capsule and the adenoma. However, this procedure is challenging when the patient has severe prostate inflammation that causes the edge to be difficult to identify, and mechanical dissection may lead to rupture of the vessel between the hyperplastic gland and the capsule in this case. When bleeding occurs, auxiliary TURP is often needed for hemostasis because the hemostatic effect of TURP (plane to point) is superior to that of laser devices (point to point). In addition, the external urethral sphincter may suffer excessive squeezing during blunt separation of the apex of the prostate, which may result in temporary or permanent impairment of muscle and postoperative SUI.

In contrast to the blunt enucleation mode of GLEP, we designed an innovative energy-based (sharp) procedure called photoselective sharp enucleation of the prostate (PSEP) with a front-firing 532-nm laser [11]. The technique combines the excellent hemostatic property of the 532-nm laser and the advantages of enucleation with high efficiency, a high tissue removal rate, and a low incidence of capsular perforation and postoperative stress urinary incontinence. To further investigate the efficacy and safety of the innovative technique, we conducted a randomized prospective controlled trial to compare the efficacy and safety of PSEP with PVP in the treatment of BPH.

## Methods

### Ethics

This study was performed in compliance with the ethical principles of the World Medical Association Declaration of Helsinki and was approved by the Ethics Committee of the First Affiliated Hospital of Army Medical University, PLA (No. KY201819). The protocol was registered in China’s clinical trial registry (No. ChiCTR1800015867).

### Patients

From June 2018 to April 2019, 179 patients with symptomatic BPH in our center were assessed for eligibility, and a total of 154 consecutive patients were enrolled in this study. All patients received preoperative examinations, including transrectal ultrasound, standard urodynamic testing, digital rectal examination, and serum prostate-specific antigen (PSA) measurement. For patients using anticoagulant/antiplatelet drugs, medications did not need to be stopped if the coagulation function (international normalized ratio) was normal. For patients with abnormal coagulation function, anticoagulant/antiplatelet drugs were discontinued for 5 days, and low-molecular-weight heparin sodium was used instead until the day before surgery. All subjects signed an informed consent form.

### Inclusion and exclusion criteria

#### Inclusion criteria

(1) Subjects older than 50 years; (2) Subjects diagnosed with lower urinary tract symptoms due to BPH. 3.Subjects had an international prostate symptom score (IPSS) > 12, maximum flow rate (Qmax) < 15 ml/s, and prostate volume (PV) > 30 ml measured at the baseline visit; 4. Subjects had indications for surgery; 5. Subjects were willing to be randomized; 6. Subjects were able to complete the examinations and self-administered questionnaires required for this trial.

#### Exclusion criteria

 (1) Subjects with other diseases who could not tolerate anesthesia during surgery; (2) Subjects with coagulopathy; (3) Subjects with malignant tumor(any malignant tumor diagnosed); (4) Subjects diagnosed with a urethral stricture (US); (5) Subjects with bladder detrusor weakness; (6) Subjects with an active infection leading to bladder urethral dysfunction; (7) Subjects with neurological disease affecting bladder function.

### Randomization method

This study was a prospective, single-center, randomized controlled clinical trial. Patients were randomized in a 1:1 ratio to either the PSEP group or PVP group using a random number table.

### Surgery

All of the operations were performed by two experienced urologists in our center (WH Shen and ZS Zhou).

#### Surgical instruments

The following instruments were used: 26 F resectoscope with a 30° lens and internal laser sheath (Olympus Medical Systems, Hamburg, Germany), a surgical green-light laser system (Aurora 160-W laser; Realton Medical Corp., Beijing, China) with a front-firing (for PSEP) or side-firing (for PVP) surgical optical fiber, and a tissue morcellator (HAWK, Hangzhou, China) with a vacuum aspiration system for PSEP.

#### Surgical methods

 The surgical method for PSEP was detailed in our previous report [11]. PVP was performed using the traditional method as reported in the literature [12].

### Data collection and follow-up

Preoperative data included IPSS, quality of life score (QoL), Qmax, PV, postvoid residual volume (PVR), PSA, and preoperative use of α-blockers, 5α-reductase inhibitors, and anticoagulants/antiplatelet drugs. Perioperative data included operative time(the time enucleating prostate), energy applied, capsular perforation, conversion to TURP for hemostasis. Patients were then interviewed 1, 6, and 12 months after surgery. The assessments included the IPSS, QoL, Qmax, PVR, and PSA at 1, 6, and 12 months postoperatively, as well as the PV at 6 and 12 months postoperatively. Perioperative and postoperative complications were recorded and analyzed using modified Clavien classification system (CCS).IPSS was the primary outcome measure, other measures were secondary. All data was collected in First Affiliated Hospital to Army Medical University.

### Statistical analysis

Statistical analysis was performed using SPSS v.23.0 (IBM Corp., Armonk, NY, USA). Continuous variables are expressed as medians and interquartile ranges. Parametric continuous variables were analyzed by Student’s t test, and nonparametric continuous variables were analyzed by Mann-Whitney U tests or Wilcoxon signed-rank tests. Categorical variables are described by frequency and percentage and were analyzed by the chi-squared test or Fisher’s exact test. For all comparisons, the assessment was considered statistically significant at the 5% level.

**Fig. 1:**
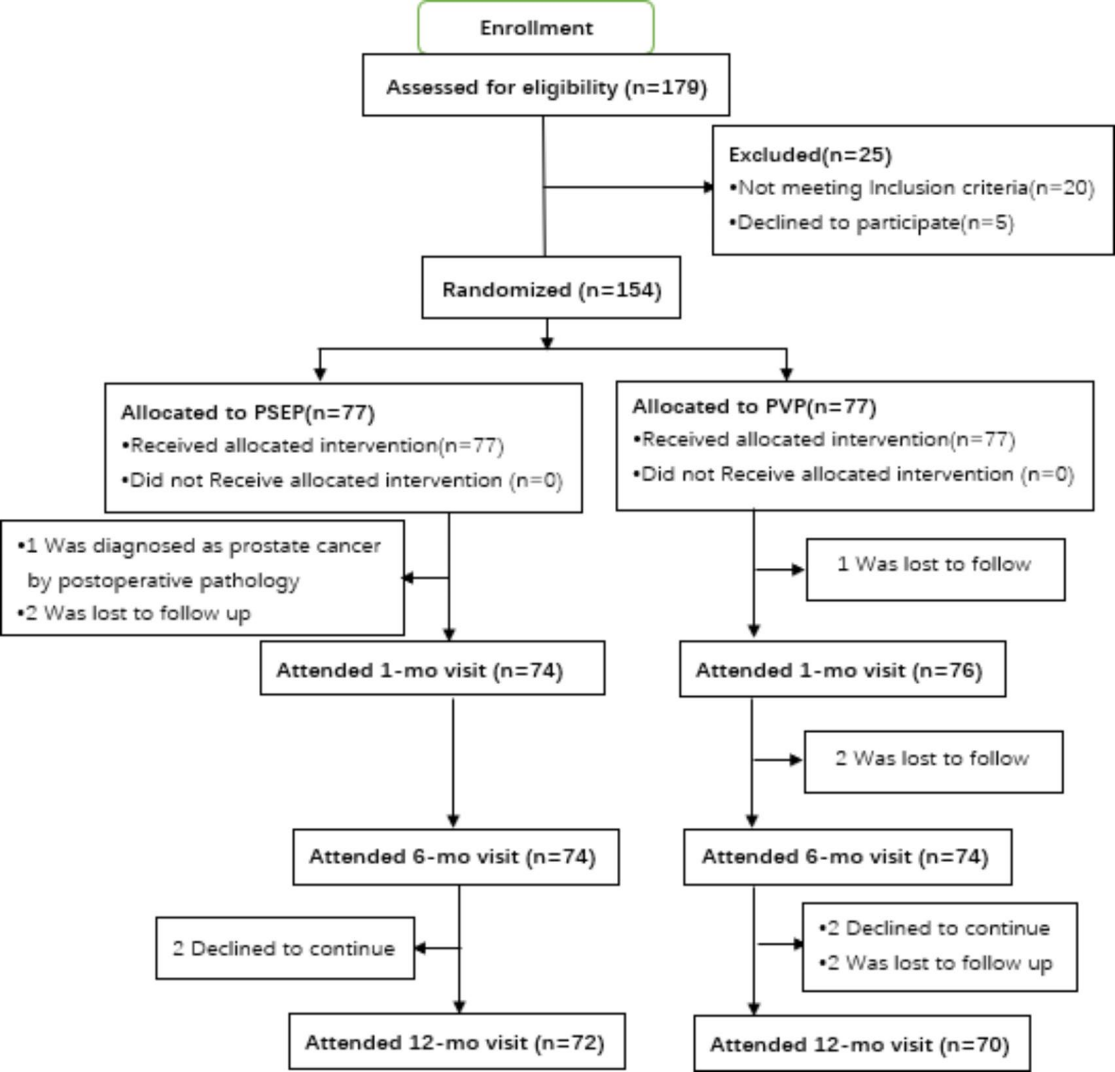
CONSORT flowchart for study participants

## Results

Figure 1 shows a flow diagram of participant progress through the trial phases. All patients who took oral anticoagulant/antiplatelet drugs had normal blood coagulation function, and medications were not stopped in the perioperative period.Bladder stones were present in 5 patients of the PSEP group (6.5%) and 6 patients of the PVP group(7.8%) ,and all were simultaneously treated with holmium laser lithotripsy.

### Baseline characteristics

**Fig. 2:**
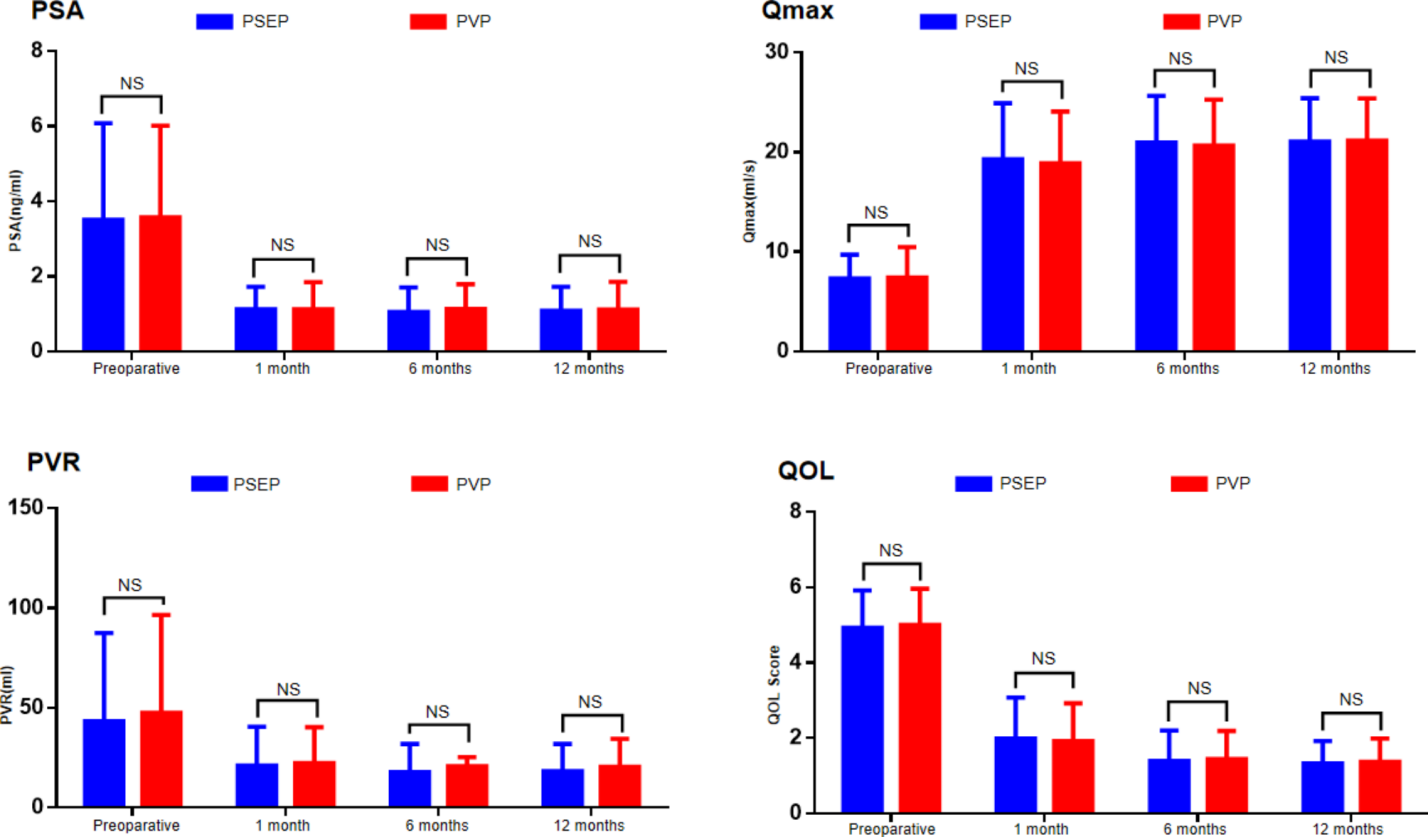
Baseline and follow-up results of PSA,Qmax,PVR and QOL(error bars indicate SD); NS = no significance

**Fig. 3:**
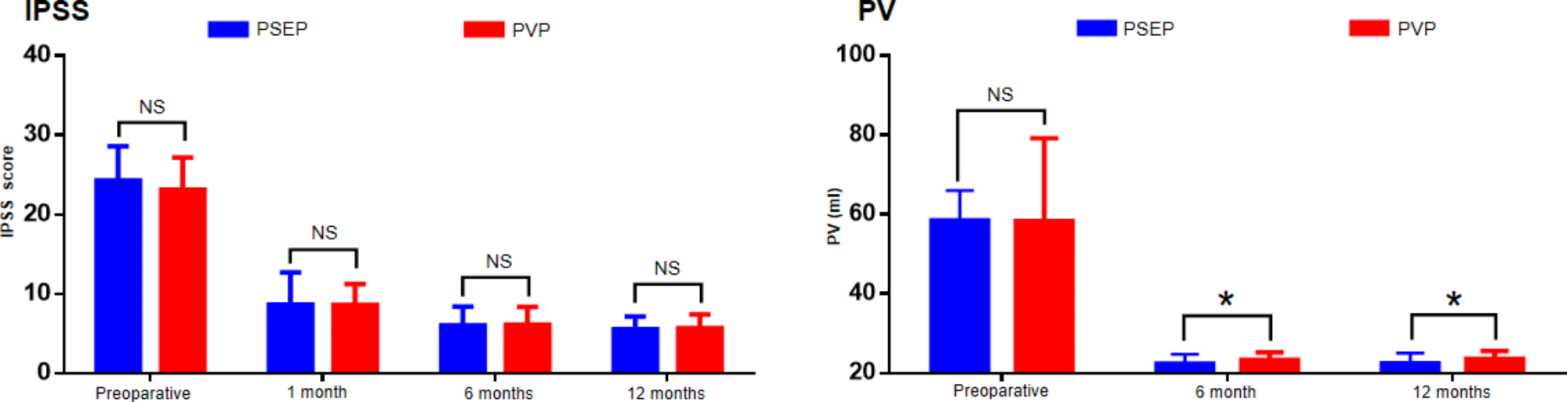
Baseline and follow-up results of IPSS and PV(error bars indicate SD); NS = no significance; *=P < 0.05

The baseline characteristics of the two groups were comparable ( Table 1 ). No significant difference was found in the baseline of Qmax, PVR, IPSS, PSA, PV and QoL of the two groups ( Figs. 2 and 3 ).

**Table 1:** The preoperative and Perioperative data of two groups

Parameter	PSEP(n = 77)	PVP(n = 77)	P value
age(years)	69(64,75.5)	70(63.5,74.5)	0.817
No.Hypertension history(%)	18(23.4%)	15(19.5%)	0.556
No.Diabetes history(%)	6(7.8%)	9(11.7%)	0.415
No.Antiplatelet/anticoagulant (%)	6(7.8%)	4(5.2%)	0.744
No.a-Blocker(%)	33(42.9%)	37(48.1%)	0.517
No.5a-reductase inhibitor(%)	18(23.4%)	21(27.3%)	0.578
Operative time (min)	35(28.5,46.5)	47(34,61)	< 0.001
Energy applied (kJ)	118(89.5,149.5)	259(197.5,337)	< 0.001
No.conversion to TURP for hemostasis	2(2.6%)	5(6.5%)	0.439
No.Capsular perforation	2(2.6%)	2(2.6%)	1

Continuous variables are expressed as medians (interquartile range);qualitative variables are expressed as frequencies(percentages)

### Perioperative data

The perioperative data of the two groups are shown in Table 1. No bladder injury occurred in any patient. The median operative time of the PSEP group was significantly shorter than that of the PVP group (35 vs. 47 min, P < 0.001), and the median energy applied in the PSEP group was significantly lower than that of the PVP group (118 vs. 259 kJ, P < 0.001). There was no significant difference in the rate of capsule perforation or auxiliary TURP for hemostasis (P > 0.05).

### Follow-up results

The follow-up results of the two groups are shown in Figs. 2 and 3.The Qmax, PVR, IPSS, and QoL of the two groups were significantly improved compared to the preoperative assessment at 1, 6, and 12 months postoperatively (P < 0.001), and no significant difference was found between the two groups (P > 0.05). At 6 and 12 months after surgery, The reduction in PV and PSA was comparable between the two groups(P > 0.05).However,the median PV At 6 and 12 months in the PSEP group was smaller than that in the PVP group (P < 0.05) (Fig. 3).

### Complications

Postoperative complications of the two groups are listed in Table 2. There was no significant difference in the occurrence of various postoperative complications between the two groups (P > 0.05). One patient in the PSEP group and two patients in the PVP group developed stress urinary incontinence (SUI) after surgery and recovered within 1 month after the initiation of pelvic floor muscle exercises. Seven patients in the PSEP group and six patients in the PVP group developed acute urinary retention after catheter removal, resulting in no significant difference between the two groups, and the patients were able to void normally 1–2 weeks after the catheter was replaced. Postoperative bladder neck contracture (BNC) occurred in two patients in the PSEP group and four patients in the PVP group. One patient in the PSEP group and two patients in the PVP group underwent bladder neck incision-resection plus regular urethral dilatation, while the remaining patients recovered after regular urethral dilation. Six patients in the PSEP group and five patients in the PVP group developed US, and all of these patients recovered after regular urethral dilatation. Six patients in the PSEP group and eight patients in the PVP group developed postoperative hemorrhage. Four patients in the PSEP group and six patients in the PVP group improved after catheter balloon dilation and continuous bladder irrigation, whereas conservative treatment failed in two patients in each group who underwent a secondary surgery for hemostasis.

**Table 2:** Complications of two groups

	0–1月			1–6月			6–12月	
Events	PSEP	PVP		PSEP	PVP		PSEP	PVP
Clavien-Dindo Grade I								
Bleeding	4	6		0	0		0	0
Postoperative bladder spasm	10	10		0	0		0	0
Urethral stricture	4	3		2	2		0	0
Bladder neck contracture	0	0		0	2		1	0
Stress urinary incontinence	1	2		0	0		0	0
Urinary retention	7	6		0	0		0	0
Clavien-Dindo Grade II								
Urinary tract infection	7	9		1	2		0	0
Clavien-Dindo Grade IIIa								
Bladder neck contracture	0	0		1	1		0	1
Bleeding	2	2		0	0		0	0
Total^#^	25	28		4	7		1	1

#The total number of patients does not equal the total number of events because some patients had multiple complications

## Discussion

The study is a further investigation of the efficacy and safety of PSEP, which was reported as an innovative technique in our previous retrospective study. PSEP achieved “energy-based” enucleation by using a front-firing 532-nm laser, not only completely removed the hyperplastic glands, but also took full advantage of the hemostasis benefits of the laser.

The rates of auxiliary TURP for hemostasis in the two groups (2.6% vs. 6.5%) were lower than those reported for GLEP (10–46%) [6, 9, 10], and the rates of capsular perforation in the two groups (2.6% vs. 2.6%) were also lower than those reported for GLEP (11.6–28%) [6, 9]. The rate of postoperative SUI was low in both the PSEP group (n = 1, 1.3%) and the PVP group (n = 2, 2.6%), and all patients recovered within 1 month after surgery by performing pelvic floor muscle exercises. The incidence of SUI is related to surgical experience and the operative technique [6, 8, 13]. According to our experience, the occurrence of SUI is associated with improper management of the apex of the prostate, where the external urethral sphincter is located. An approximately 5-mm distance from the proximal verumontanum at the 11 to 1 o’clock position of the apex is maintained in the PSEP technique through convenient and accurate management with the front firing fiber. This procedure plays a positive role in postoperative urinary control and may be the main reason for the low rate of SUI in the PSEP group. The above advantages benefit from the avoidance of mechanical separation with the beak of the scope.

The improvements in IPSS, QoL, PVR, and Qmax were comparable between the two groups, suggesting that the methods used in both groups were effective in the treatment of BPH. PSEP has several advantages compared with PVP. First, it has a higher efficiency. Heat-induced fiber degradation and loss of power output of a side-firing fiber due to the presence of a refraction device during PVP remain problems that cannot be ignored. Hermanns et al. showed that the median output power of the side-firing fiber decreased to 77%, 57%, and 51% of the initial value after the application of 25, 150, and 250 kJ [14]. PSEP avoided the above problems by using a top-firing fiber without a refractive device. In addition, enucleation avoided coagulated seam development, which occurred during the layer-by-layer vaporization of PVP and significantly increased the efficiency of the operation. These are the main reasons why the median operative time and median energy applied in the PSEP group were lower than those in the PVP group in our study. Second, PSEP can remove more hyperplastic tissue than PVP. The limited vaporization depth of PVP often results in difficulty reaching the capsule due to the thick coagulated seam, whereas PSEP can completely remove the hyperplastic tissue along the capsule. This might be the main reason why the PV was smaller in the PSEP group than in the PVP group at 6 and 12 months after surgery in this study. Third, PSEP retains tissue specimens and reduces the risk of missed diagnosis of prostate cancer. A study by Bierset al. showed that TURP-detected prostate cancers accounted for 1.5–5.6% of all newly diagnosed prostate cancers per year [15], and one patient (1.3%) treated for BPH in our center was diagnosed with prostate cancer after PSEP. Therefore, the lack of tissue specimens after PVP is still a disadvantage that cannot be ignored.

PSEP is also safe and effective for patients taking oral antiplatelet/anticoagulant drugs. None of the ten patients (six in the PSEP group and four in the PVP group) who received antiplatelet/anticoagulant drugs in this study received blood transfusion or reoperation for hemostasis during the perioperative period, which is in line with previous results [2].

The incidence of US and BNC reported in the literature exhibits a wide range. In our study, the incidence rates of US at 12 months after the operation in the PSEP and PVP groups were 7.8% (6/77) and 6.5% (5/77), respectively, and the incidence rates of BNC were 2.6% (2/77) and 5.2% (4/77). These rates are consistent with previous reports [16, 17]. The exact etiologies for these two complications are not completely known, and many factors may be involved, including slower resection speed, intraoperative urethral mucosa rupture, postoperative continuous infection, diameter of the instrument, presence of chronic prostatitis, repeated drainage of the bladder and prostate size [16–18]. Patients with BNC in this study had a smaller PV (32–46 ml), which was similar to previous reports [18]. No other specific cause of US and BNC was found.

No patients in the PSEP group in this study suffered bladder injury due to the top-firing fiber, it was accordance with the result of our previous study. The safety of the top-firing fiber was demonstrated again.

The main limitation of this study is the relatively short follow-up time. However, 1 year of follow-up seems acceptable considering that most complications occur within 6 months after the operation according to previous experience. In addition, the small sample size was another limitation of this trial. Therefore, a multicenter randomized trial with a larger number of cases and a longer follow-up is encouraged in future studies.

## Conclusion

Both PSEP and PVP can achieve good efficacy and safety in the treatment of BPH. PSEP can remove more tissue than PVP and is associated with higher efficiency. In addition, PSEP eliminates the problem of lack of tissue samples associated with PVP and may reduce the risk of missed diagnosis of prostate cancer.

## Data Availability

The datasets generated and analysed during the current study are not publicly available due to protect study participant privacy but are available from the corresponding author on reasonable request.

## Abbreviations

BNC: Bladder neck contracture.
BPH: Benign prostatic hyperplasia.
CCS: Clavien classification system.
GLEP: GreenLight laser enucleation of the prostate.
HoLEP: Holmium laser enucleation of the prostate.
IPSS: International prostate symptom scor.
PSA: Serum prostate specific antigen.
PSEP: Photoselective sharp enucleation of the prostate.
PV: Prostate volume.
PVP: Photoselective vaporization of the prostate.
PVR: Post-void residual urine.
PVRP: Photoselective vaporesection of the prostate.
QoL: Quality of Life score.
Qmax: Maximum flow rate.
SUI: Stress urinary incontinence.
TURP: Transurethral resection of the prostate.
US: Urethral stricture.

## References

[1] Lai S, Peng P, Diao T, Hou H, Wang X, Zhang W, Liu M, Zhang Y, Seery S, Wang J. Comparison of photoselective green light laser vaporisation versus traditional transurethral resection for benign prostate hyperplasia: an updated systematic review and meta-analysis of randomised controlled trials and prospective studies. BMJ Open 2019, 9(8).10.1136/bmjopen-2018-028855. 10.1136/bmjopen-2018-028855 PMC6707662 31439603

[2] Eken A, Soyupak B (2018). Safety and efficacy of photoselective vaporization of the prostate using the 180-W GreenLight XPS laser system in patients taking oral anticoagulants. J Int Med Res.

[3] Guo S, Muller G, Lehmann K, Talimi S, Bonkat G, Puschel H, Gasser T, Bachmann A, Rieken M (2015). The 80-W KTP GreenLight laser vaporization of the prostate versus transurethral resection of the prostate (TURP): adjusted analysis of 5-year results of a prospective non-randomized bi-center study. Lasers Med Sci.

[4] Valdivieso R, Hueber PA, Meskawi M, Belleville E, Ajib K, Bruyere F, Te AE, Chughtai B, Elterman D, Misrai V (2018). Multicentre international experience of 532-nm laser photoselective vaporization with GreenLight XPS in men with very large prostates. BJU Int.

[5] Castellani D, Pirola GM, Rubilotta E, Gubbiotti M, Scarcella S, Maggi M, Gauhar V, Teoh JY, Galosi AB (2021). GreenLight Laser Photovaporization versus Transurethral Resection of the Prostate: A Systematic Review and Meta-Analysis. Res Rep Urol.

[6] Misrai V, Kerever S, Phe V, Zorn KC, Peyronnet B, Roupret M (2016). Direct Comparison of GreenLight Laser XPS Photoselective Prostate Vaporization and GreenLight Laser En Bloc Enucleation of the Prostate in Enlarged Glands Greater than 80 ml: a Study of 120 Patients. J Urol.

[7] Stone BV, Chughtai B, Forde JC, Tam AW, Lewicki P, Te AE (2016). Safety and Efficacy of GreenLight XPS Laser Vapoenucleation in Prostates Measuring Over 150 mL. J Endourol.

[8] Misraï V, Pasquie M, Bordier B, Elman B, Lhez JM, Guillotreau J, Zorn K (2018). Comparison between open simple prostatectomy and green laser enucleation of the prostate for treating large benign prostatic hyperplasia: a single-centre experience. World J Urol.

[9] Panthier F, Pasquier J, Bruel S, Azancot V, De La Taille A, Gasman D (2019). En bloc greenlight laser enucleation of prostate (GreenLEP): about the first hundred cases. World J Urol.

[10] Elshal AM, Elkoushy MA, El-Nahas AR, Shoma AM, Nabeeh A, Carrier S, Elhilali MM (2015). GreenLight laser (XPS) photoselective vapo-enucleation versus holmium laser enucleation of the prostate for the treatment of symptomatic benign prostatic hyperplasia: a randomized controlled study. J Urol.

[11] Wang Y, Liu Z, Jiang T, Zhou X, Chen Z, Zheng J, Yan D, Zhou Y, Zhou Z, Shen W (2021). Photoselective sharp enucleation of the prostate with a front-firing 532-nm laser: an innovative surgical technique for benign prostatic hyperplasia-a single-center study of 475 cases. World J Urol.

[12] Bachmann A, Tubaro A, Barber N, d’Ancona F, Muir G, Witzsch U, Grimm MO, Benejam J, Stolzenburg JU, Riddick A (2014). 180-W XPS GreenLight laser vaporisation versus transurethral resection of the prostate for the treatment of benign prostatic obstruction: 6-month safety and efficacy results of a European Multicentre Randomised Trial–the GOLIATH study. Eur Urol.

[13] Endo F, Shiga Y, Minagawa S, Iwabuchi T, Fujisaki A, Yashi M, Hattori K, Muraishi O (2010). Anteroposterior dissection HoLEP: a modification to prevent transient stress urinary incontinence. Urology.

[14] Hermanns T, Grossmann NC, Wettstein MS, Keller EX, Fankhauser CD, Gross O, Kranzbuhler B, Luscher M, Meier AH, Sulser T, et al: **Is loss of power output due to laser fiber degradation still an issue during prostate vaporization using the 180 W GreenLight XPS laser?** *World Journal of Urology* 2019, **37**(1):181–187.10.1007/s00345-018-2377-5. 10.1007/s00345-018-2377-5 29923013

[15] Biers SM, Oliver HC, King AJ, Adamson AS (2009). Does laser ablation prostatectomy lead to oncological compromise?. BJU Int.

[16] Primiceri G, Castellan P, Marchioni M, Schips L, Cindolo L (2017). Bladder Neck Contracture After Endoscopic Surgery for Benign Prostatic Obstruction: Incidence, Treatment, and Outcomes. Curr Urol Rep.

[17] Grechenkov A, Sukhanov R, Bezrukov E, Butnaru D, Barbagli G, Vasyutin I, Tivtikyan A, Rapoport L, Alyaev Y, Glybochko P (2018). Risk factors for urethral stricture and/or bladder neck contracture after monopolar transurethral resection of the prostate for benign prostatic hyperplasia. Urologia.

[18] Tao H, Jiang YY, Jun Q, Ding X, Jian DL, Jie D, Ping ZY (2016). Analysis of risk factors leading to postoperative urethral stricture and bladder neck contracture following transurethral resection of prostate. Int Braz J Urol.

