# Correction to: Photoselective sharp enucleation of the prostate with a front-firing 532-nm laser versus photoselective vaporization of the prostate in the treatment of benign prostatic hyperplasia: a randomised controlled trial with 1-year followup results

**DOI:** 10.1186/s12894-022-01154-w

**Published:** 2022-12-10

**Authors:** Zhengchao Liu, Zhipeng Chen, Dishi Yan, Tao Jiang, Jian Fu, Jun Zheng, Yuanxiu Zhou, Zhansong Zhou, Wenhao Shen

**Affiliations:** 1 Urological Institution of the People’s Liberation Army, First Affiliated Hospital to Army Medical University, Third Military Medical University, Chongqing, 400037 China; 2 Department of Anesthesiology, Daping Hospital, Third Military Medical University, Chongqing, China

**Correction to: BMC Urology (2022) 22:173** 10.1186/s12894-022-01129-x

Following publication of the original article [1], the authors identified that the article note about equal contribution was missing.

The first and second author, Zhengchao Liu and Zhipeng Chen, contributed equally to the article.

It has been added in this correction.

The original article has been updated.
